# Sliding walls: a new paradigm for fluidic actuation and protocol implementation in microfluidics

**DOI:** 10.1038/s41378-019-0125-7

**Published:** 2020-04-06

**Authors:** Bastien Venzac, Yang Liu, Ivan Ferrante, Pablo Vargas, Ayako Yamada, Rémi Courson, Marine Verhulsel, Laurent Malaquin, Jean-Louis Viovy, Stéphanie Descroix

**Affiliations:** 1 Laboratoire Physico Chimie Curie, Institut Curie, PSL Research University, CNRS UMR168, 75005 Paris, France; 2 Sorbonne Universités, UPMC Univ Paris 06, 75005 Paris, France; 3 Institut Pierre-Gilles de Gennes, PSL Research University, 75005 Paris, France; 4 Institut Curie, PSL Research University, CNRS UMR 144, 75005 Paris, France; 5 LAAS-CNRS, Université de Toulouse, CNRS, 3, 1400 Toulouse, France

**Keywords:** Engineering, Chemistry, Engineering, Chemistry, Engineering

## Abstract

Currently, fluidic control in microdevices is mainly achieved either by external pumps and valves, which are expensive and bulky, or by valves integrated in the chip. Numerous types of internal valves or actuation methods have been proposed, but they generally impose difficult compromises between performance and fabrication complexity. We propose here a new paradigm for actuation in microfluidic devices based on rigid or semi-rigid walls with transversal dimensions of hundreds of micrometres that are able to slide within a microfluidic chip and to intersect microchannels with hand-driven or translation stage-based actuation. With this new concept for reconfigurable microfluidics, the implementation of a wide range of functionalities was facilitated and allowed for no or limited dead volume, low cost and low footprint. We demonstrate here several fluidic operations, including on/off or switch valving, where channels are blocked or reconfigured depending on the sliding wall geometry. The valves sustain pressures up to 30 kPa. Pumping and reversible compartmentalisation of large microfluidic chambers were also demonstrated. This last possibility was applied to a “4D” migration assay of dendritic cells in a collagen gel. Finally, sliding walls containing a hydrogel-based membrane were developed and used to concentrate, purify and transport biomolecules from one channel to another, such functionality involving complex fluidic transport patterns not possible in earlier microfluidic devices. Overall, this toolbox is compatible with “soft lithography” technology, allowing easy implementation within usual fabrication workflows for polydimethylsiloxane chips. This new technology opens the route to a variety of microfluidic applications, with a focus on simple, hand-driven devices for point-of-care or biological laboratories with low or limited equipment and resources.

## Microfluidics: Novel technology for microfluidic device operation—“sliding walls”

A new technology, termed “sliding walls,” has been developed for operating microfluidic devices (which function at sub-millimetre scales), whereby rigid or semi-rigid walls, hundreds of micrometres thick, slide within a microfluidic chip. Fluid control in micro-devices is currently achieved largely by expensive, bulky external valves and pumps or by valves integrated in the chip. Such internal valves or actuation methods generally have shortcomings related to performance and complex fabrication. A team headed by Stephanie Descroix and Jean-Louis Viovy at the Institut Curie—Institut Pierre Gilles de Gennes, Paris applied its sliding wall technique and was able to execute several fluidic operations in which channels could be blocked or reconfigured through the sliding wall geometry. The authors believe that their technology has the potential for various microfluidic operations in biological laboratories with limited equipment and resources.

## Introduction

In microfluidics, truly reconfigurable systems remain an engineer’s dream. “Reconfigurable” is often used to describe clever systems built in modular units that can be assembled, allowing for a quick reorganisation of the channel network between experiments^1–3^. However, for most microfluidic systems, the whole channel network is fixed during the microfabrication steps and cannot be reconfigured on-demand during experiments. In most cases, the only possibilities for users are limited to changes in pumping (moving fluids in, out and inside the device), valving (blocking different compartments in the device) or using external forces (electric, magnetic^4^, optic^5^, acoustic^6^, etc.).

While fully reconfigurable systems still do not exist, the integration of pumps and valves inside microfluidic chips extends the fluidic actuation possibilities. A large variety of microfluidic valves have been developed, the most famous being pinch valves based on the work of Quake’s group^7^. This strategy involves the deflection of an elastomeric membrane separating a flow and a control channel in a two-level cross configuration by pressure in the control channel. This concept has led to commercial products, and the integration of a large amount of valves (up to 1 million valves per cm^2^)^8^ was the closest attempt to reach a truly reconfigurable system. However, this approach requires an elaborate fabrication technology and external pressure controllers. Other approaches based on light or heat-responsive fluids or hydrogels^9,10^ or mechanical displacement via motors^11–13^ have also been developed. These systems have been successful in applications in which complex, multi-step protocols are required, and small quantities of reagents are available. However, they often require bulky, expensive external equipment (pressure-based pumps^14^, current generators, light sources, etc.), which greatly increases their footprint, price and complexity. A large quantity of applications only requires simple reconfiguration of a microfluidic network, and simpler actuation methods that do not require the acquisition of expensive equipment would allow the adoption of microfluidic systems for applications in which size, portability, simplicity of use and cost are critical, especially for non-specialists and biological laboratories in limited-resource settings^15^.

To answer such needs, manually driven or passive actuation methods have been developed. Passive pumping of liquid has been shown using capillary force in hydrophilic channels^16^ or paper^17^. These methods, however, are not reversible and operate at a single flow speed. 3D-printed pumping lids^18^ or direct displacement of liquid using finger-powered membranes^19^ allows for equipment-free and more complex actuations. In thermoplastics, screws moving in threaded ports by homemade microcontrollers have been used as integrated syringe pumps^20^. However, the integration of these methods in existing systems could be complex or impossible, limiting their adoption. Several examples of manually operated pinched valves have also been demonstrated in PDMS devices, but microfabrication was not straightforward^21,22^.

Finally, it should be noted that an interesting alternative to the above-mentioned technologies is to combine fluid transport and compartmentalisation in microfluidics as proposed by the “slip chip” concept^23^. This device uses two plastic plates in close contact. The bottom plate contains wells, which are preloaded with reagents, and the top plate comprises grooves connecting the wells of the bottom plate. By sliding the top plate onto the bottom plate, the connections between wells can be closed and opened. Highly parallelised fluidic operations can be performed with a simple mechanical motion, but the variety of operations permitted is limited, and the tight seal between the plates requires a thin lubricating layer of oil.

**Fig. 1:**
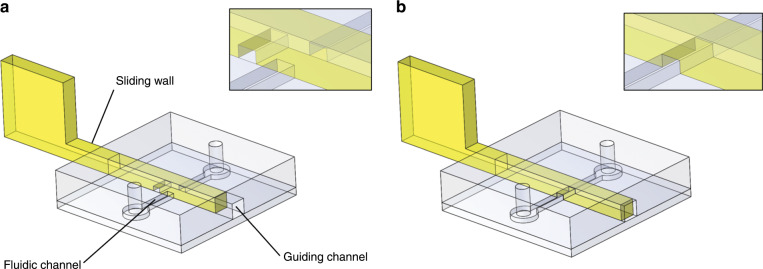
Sliding wall principle. PDMS structures contain a guiding channel and a fluidic channel and were bonded to a planar PDMS surface. In this example, a sliding wall with an engraved channel was inserted after chip fabrication inside the guiding channel. The fluidic channel was **a** blocked or **b** free. Details of the sliding wall/fluidic channel intersection are provided in the inserts.

Here, we propose a new concept for microfluidic actuation, called “sliding walls”, which (i) is compatible with usual soft-lithography fabrication; (ii) does not require external equipment; (iii) can be manually operated; and (iv) allows integration in a single component of a combination of valving, molecule purification, concentration or extraction. Briefly, sliding walls, i.e., rigid or semi-rigid structures with transversal dimensions of hundreds of micrometres were created with several manufacturing methods (casting in PDMS moulds, 3D printing or micro-milling). They were pushed in open-end channels in PDMS chips and longitudinally displaced (see Fig. 1). This actuation permitted reversible opening or closing of a channel, pumping of fluids, reorienting flows, and generally reconfiguring a microfluidic network at will. The sliding walls could also be engraved and comprise ducts, holes or windows, allowing the transport of molecules, species or fluid aliquots from one channel to another. We describe here the principle of the method and demonstrate simple functions, such as valving and pumping, along with more advanced functions, including (i) the formation of hydrogel slabs for “4D” controlled cell culture and (ii) membrane-based electrokinetic DNA preconcentration combined with buffer exchange. We demonstrate the possibility of implementing this technology at low cost for fast prototyping. This first-generation family uses manual actuation of the sliding wall for simplicity; however, these sliding walls could also be driven by computer-controlled micro-motors or actuators for full automation. Altogether, this new toolbox is especially well adapted to applications involving channel dimensions above 100 µm and requires few actuation elements without the need for expensive and bulky external equipment.

## Results and discussion

### Sliding wall fabrication principle

The general principle of sliding wall technology is the following: a rigid or semi-rigid structure is inserted into a guiding channel open in the PDMS microfluidic chip sides (see Fig. 1). This channel intersects the fluidic channels, and the translation of the rigid structure inside the guiding channel modifies the fluidic network.

Sliding wall fabrication can be performed using a large variety of usual fabrication methods and materials. To demonstrate that, we used here: (i) stainless-steel films shaped in planar sliding walls by micro-milling; (ii) photocurable resist (NOA 81) photopolymerised in PDMS moulds; and (iii) photocurable resin (DS3000) shaped by stereolithographic 3D printing (see SI for details). The fabrication methods/materials were chosen for each subsequent experiment according to their intrinsic properties. Miniaturisation of a sliding wall is limited by the rigidity of the material to avoid wall buckling and/or breaking during actuation. When miniaturising a sliding wall, the risk of buckling and damage increases. Imagine a wall with a square section (lateral dimension l) inserted by a length L inside the guiding channel. Friction on this inserted length scales as L × l. The critical force before buckling scales as l^4^. Equilibrating the friction and the buckling force, we obtain that the critical inserted length before buckling scales as l^3^. Sliding walls with a smaller section start to buckle with a shorter length inserted in the guiding channel. The observed minimal thickness of the sliding walls for convenient operation without wall damage was 100 µm with stainless steel and 500 µm with NOA 81 and 3D-printed resin. Stainless steel is thus preferred when thin sliding walls are needed (compartmentalisation experiments).

For large sliding walls (section of 1.2 mm × 1.2 mm for the pumping experiment), conventional stereolithography has been used, while high-precision 3D-printing and NOA81 casting were necessary for sliding walls with sections of 500 µm (valves and electrokinetic preconcentration) because of the higher resolution and smaller roughness of these techniques. Finally, the smallest features integrated on a sliding wall (50 × 100 µm ducts) used for the switching valve were produced in our facilities by micro-milling on stainless steel.

### Valves

**Fig. 2:**
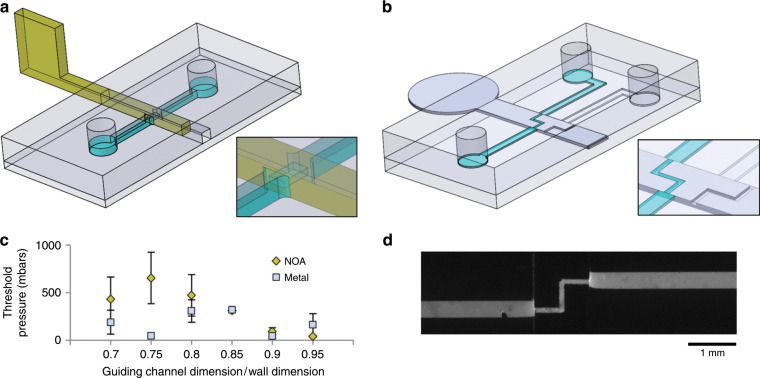
Valving experiments. **a** Design of the chip and NOA-based sliding wall for the on-off valve experiment. **b** Design of the chip and the metallic sliding wall for the switch valve experiment. **c** Maximum pressure withstood by NOA-based (yellow series) and metal-based walls (grey series) for different ratios between the guiding channel and sliding wall heights and widths (three experiments per condition). **d** Fluorescent image of the switch valve with fluorescein-laden water flowing through the open path (13 µl/s).

As an initial proof-of concept of this reconfigurable technology, we prepared two types of valves: a NOA-based on/off valve (Fig. 2a) and a metallic switch valve with one inlet and two outlets (Fig. 2b). The NOA-based sliding wall had a 500 µm × 500 µm section with a window (section 100 µm × 250 µm) perpendicular to the wall axis (see SI). The micro-milled stainless-steel sliding wall (section 100 µm × 950 µm) had integrated ducts (section 50 µm × 100 µm) engraved on the surface of the structure. To ensure good sealing of the fluidic channels in the off position and to avoid leakage along the guiding channel, the cross-section of the guiding channel must be slightly smaller than the cross-section of the sliding wall. The optimum ratio between these two sections was experimentally optimised for both valves, and the maximum pressure withstood by the valves in the off position before leakage was measured (see SI). For ratios of 95% and 90%, leakage appeared below an applied pressure of 5 kPa (Fig. 2c), while for a ratio of 85%, both valves reproducibly withstood 30 kPa (300 mbar) of pressure (*n* = 3). For guiding channels of smaller sections (ratios ranging from 80% to 70%), larger pressures could be withstood by the NOA-based wall (with a maximum over 90 kPa (900 mbar), but at the price of lower reproducibility. This lack of reproducibility at high pressure could be explained by the higher probability of damaging the guiding channel when introducing the sliding wall by tearing PDMS fragments or by delaminating the PDMS-PDMS bonding. This range of pressure for safe actuation would make the technology poorly suited for applications requiring high pressures (in the range of 1 bar and above), for instance applications relying on inertial effects (which requires high flow speeds, in the range of m/s, and thus high pressure drops), or microfluidic networks with a high hydrodynamic resistance (e.g., networks of channels with transversal dimensions under 10 µm or porous material). In contrast, the technology is well suited to applications involving channels in the 100 µm range and flow speeds up to a few tens of cm/s, such as organ-on-chip or cell culture studies, or numerous analytical applications. In these systems, working in a pressure range of 0–30 kPa is highly acceptable; as an illustration, 300 mbar pushed 1 µl of water per minute in a tube of 1 cm in length and 20 µm in diameter.

The NOA-based sliding wall, manually actuated, permitted an efficient on/off actuation at most integrated valves. We then demonstrated here a more complex operation with the metallic sliding wall using two ducts micromilled on the wall: depending on the position of the sliding wall, the inlet channel was connected to one of the two outlets, and fluorescein-laden water was pushed through the valve, as shown in Fig. 2d, at a maximum flow rate of 13 µl/s for 400 mbar of pressure.

These first examples validated the good sustainable pressure range for the application of these valves and the simple manual reconfiguration of microfluidic networks. Due to their comparatively large areas compared to classical pinch valves, their manual actuation, and the fact that they do not require any external energy to remain in the selected position (either open or close), those sliding valves are particularly interesting for applications in which a few network reconfigurations are needed without any external equipment, for example organ-on-chips and cell culture devices in incubators, as demonstrated in future examples.

### Sliding pump

**Fig. 3:**
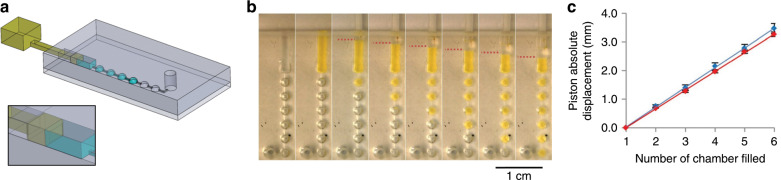
Pumping experiment. **a** Chip design. **b** Sequential pictures of the pumping of fluorescein-laden water through 1 µl chambers. Position of the piston is indicated with dashed red lines. **c** Liquid displacement versus absolute piston displacement (piston origin was set at the onset of the filling of the first chamber), for pushing (blue) then pulling (red), averaged over four different devices.

In addition to the valve functions, we also demonstrated the use of sliding walls as on-chip syringes for manual pumping of fluids. The sliding wall and microfluidic chip designs are presented in Fig. 3. For this experiment, sliding walls were fabricated by stereolithographic 3D printing (mean roughness of approximately 50 µm). The microfluidic network consisted of a large guiding chamber (section 1.5 mm × 1 mm), which was also used for lateral loading of 10 µl of fluorescein-laden water before insertion of the sliding wall. This chamber was linked to six hemispherical 1 µl chambers in series with an open outlet. The sliding wall was inserted and manually pushed, sequentially filling the chambers (Fig. 3b). The sliding wall was then pulled to empty the chambers. No leakage of liquid during the pushing or aspiration of air during the pulling was observed during these experiments performed with four different devices. The number of chambers filled is plotted against the absolute piston displacement (with the origin taken when the first chamber was filled or emptied) (Fig. 3c). Pumping is reversible as the pushing and pulling curves are superimposed within experimental error.

In these experiments, involving relatively large volumes, simple manual operation was sufficient, but of course, sliding walls can be mounted on micrometre positioning stages or actuated by micro-motors for more accurate and automated flow rate control while retaining the advantage of small dead volumes compared to classical syringe pumps.

### Large chamber compartmentalisation

The sliding walls are also an interesting tool for the compartmentalisation of large microfluidic chambers (height > 200 µm). Several approaches allowing the controlled partitioning of a chamber into several independent compartments have been proposed in the past. For example, modified pneumatic valves were able to lift and lower a wall connected to a mobile roof^24^. Our team proposed the use of a manually removable nylon wire for transient compartmentalisation^25,26^, with several applications, such as 2D cell co-culture, differential chemical patterning and 3D cell culture in hydrogel. A similar concept could be applied to sliding walls, while new applications are possible with their higher rigidity and more complex designs, e.g., creation of a temporary chemical gradient and “4D” cell culture applied to cell migration assay.

**Fig. 4:**
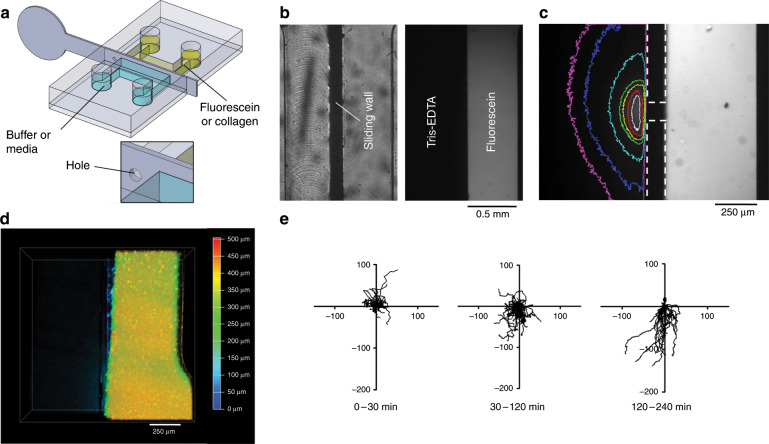
Compartmentalisation experiments. **a** Design of the chip and metallic sliding wall. **b** Top-view pictures of a sealing test. Left: bright picture of the chamber. Right: Fluorescent image of the chamber after 8 h. **c** Gradient of fluorescein in the Tris-EDTA buffer compartment after placement of a 200 µm hole in the sliding wall inside the chamber. Sliding wall and hole limits are indicated with the dotted lines. The colour lines correspond to the image surface with an intensity higher than 12% of the maximum value (white: 1 s, red: 4 s, yellow: 9 s, green: 14 s, cyan: 50 s, blue: 110 s, magenta: 170 s after wall displacement). **d** Top-view, depth-coded confocal image of a fluorescent, gelled collagen slab in the right, half bottom of the chamber after removal of the sliding wall. **e** Trajectories of dendritic cells inside the collagen slab before sliding wall removal (0–30 min) and after sliding wall removal (30–240 min) decomposed in two periods. The first one showed no preferential migration (30–120 min), while cells are attracted to the chemokine compartment from 120 to 240 min. The axes are in micrometres, and the vertical axis points away from the chemokine compartment.

For these experiments, the chamber design is shown in Fig. 4a. Two narrow grooves were added to the chamber roof and floor to guide a vertical stainless-steel sliding wall (width 100 µm, height 950 µm). A sealing test was performed by filling the right half of the chamber with fluorescein and the other side with Tris-EDTA buffer (Fig. 4b). The fluorescence intensity was followed for 8 h along the wall interface, and the intensity did not vary in the two chambers, validating the absence of leakage between the two compartments. Pulling the sliding wall out of the chamber (without removing it fully from the device) allowed full communication between the compartments. Compared to nylon wires, the sliding walls also allowed the implementation of localised delivery at will of molecules from one compartment to the second compartment. At the beginning of the experiment, a sliding wall partitioned the chamber, with fluorescein in the right compartment and buffer in the left one. A 200 µm cylindrical hole in the sliding wall (Fig. 4a) was located outside of the chamber. By sliding the wall, the hole was placed inside the chamber, and fluorescein penetrated the left compartment, creating a slowly advancing gradient (see Fig. 4c). The sliding wall was then reset at its initial position, closing the flow between the two compartments. Temporary gradients of molecules were thus achieved with precise control of the starting time and the spatial localisation and with minimal fluid displacement (<2 nl in this particular configuration).

### Hydrogel slab integration and “4D” migration assay

The spatial patterning of hydrogels into compartments with large interfaces is a major requirement for the development of 3D organ-on-chip^27^. Until recently, this was mostly achieved via the confinement of hydrogel solution by capillarity using lines of pillars^28^. Interfaces created this way, however, are neither planar nor continuous and thus are far from physiologically realistic. Alternately, the creation of large planar interfaces was demonstrated using an elegant technique with detachable PDMS lids^29^. Similar interfaces can easily be produced using sliding walls. Here, a fluorescent collagen solution was loaded in the right half of a chamber compartmentalised with a sliding wall and kept at 37 °C to induce gelation. After filling the second half with PBS buffer, the vertical sliding wall was removed. A hydrogel slab with a sharp and planar interface was obtained (height 500 µm, length 3 mm), as observed by confocal microscopy (Fig. 4d).

The potential of this technology to investigate cellular migration was demonstrated using dendritic cells loaded within a collagen solution in one half of the chamber. Dendritic cells are the antigen-presenting agents of the immune system and are able to quickly migrate towards lymphatic vessels following gradients of CCL21 chemokine. After gelling, the second compartment was filled with this chemokine solution. The removal of the sliding wall after 30 min created a straight interface between the two compartments, and the chemoattractant diffused in the collagen slab. As expected, dendritic cells migrated towards the gel/solution interface, as shown in Fig. 4e (Supplementary Movie M1). Contrary to Transwell systems, the planar configuration of these devices provided a decisive advantage for the observation and microscopy analysis of cell migration. Moreover, fabrication of chips with more than two compartments is also possible, opening the route to complex co-culture experiments, where the cells can be grown in separate compartments and put into contact by displacing sliding walls at desired time points. We call this approach the “4D” cell culture. Care must be taken concerning the biocompatibility of the materials, which is crucial for cell-based assays, especially when using 3D-printed structures. For this example, sliding walls were made with stainless steel, a typical biocompatible material used in clinics.

### Membrane-based electrokinetic DNA preconcentration and purification

The previous experiments demonstrated the potential of the sliding wall toolbox for three elementary liquid handling operations, i.e., valving, pumping, and compartmentalisation. Sliding walls, however, can also be used to perform less common operations, involving in particular flow-free transport. Here, hydrogel membranes were integrated inside sliding walls for electrokinetic preconcentration, transport and release of DNA macromolecules. Size-based trapping of molecules or cells with a permeable membrane has been previously shown in microfluidic chips^30,31^, and hydrogels were also used to pre-concentrate large molecules^32,33^. However, gel loading or polymerisation in microsystems with accurate boundaries is still not straightforward and requires tedious fabrication processes. Here, we present the first integration of movable and reconfigurable hydrogel membranes in microfluidic systems.

**Fig. 5:**
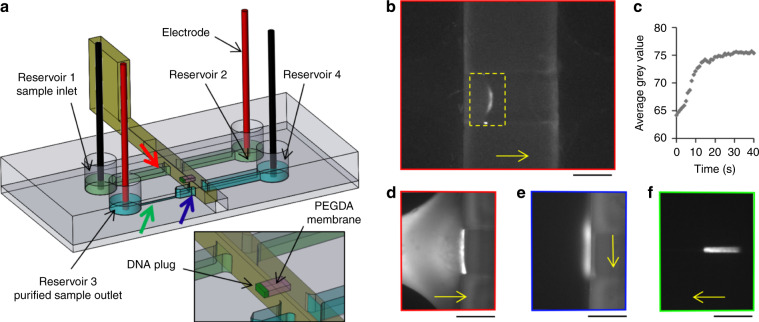
DNA preconcentration and purification experiment. **a** Design of the chip and sliding wall. A PEGDA membrane (pink) was photopolymerized in the window of a sliding wall. Coloured arrows indicate the location of the following pictures with the corresponding coloured border. **b** Preconcentration by electrophoresis of 100 pg of Lambda-DNA against the PEGDA membrane in a 3D-printed sliding wall. **c** Evolution over time of the average grey value inside the yellow rectangle of **b**). **d** Fluorescent pictures of DNA during preconcentration against the PEGDA membrane, **e** after displacement to the second channel and **f** electrophoretic release. Scale bars: 250 µm. DNA migration or displacement directions are indicated by the yellow arrows.

For this experiment, the sliding wall had a design similar to that used for valving with an integrated window (see Fig. 5a) and was produced with a high-resolution stereolithographic 3D printer^34^. A solution of polyethylene-glycol-diacrylate 700 (PEGDA) in Tris-borate-EDTA buffer (concentration 45% v/v) was photopolymerized inside the window, producing a hydrogel with a pore size of approximately 5 nm^35^, and was slid to the intersection with the fluidic channel linking reservoirs 1 and 2 (Fig. 5a). Electrophoretic migration of 48.5 kbp Lambda-DNA labelled with SyBr Green in Tris-borate-EDTA buffer, initially loaded in reservoir 1, was performed by applying a constant electric field in the channels between reservoirs 1 and 2. The hydrogel pore size was too small to allow the migration of double-stranded DNA molecules, inducing preconcentration of the DNA at the membrane.

The smallest amount of DNA detected after preconcentration was 100 pg (around the DNA content of 16 human cells), as shown in Fig. 5b. As preconcentration took place, the fluorescent signal in front of the membrane increased with a plateau after 30 s (Fig. 5c). To demonstrate the versatility of the approach and the possibility to easily use other hydrogel membranes, a similar experiment was performed with agarose hydrogel instead of PEGDA. This also led to DNA concentration, except that the DNA entered the gel due to the larger pore size of agarose compared to PEGDA (see Supplementary Fig. S10).

We also showed the ability to displace this preconcentrated plug of DNA to another channel to perform a buffer exchange step. After preconcentration of the DNA sample (Fig. 5d, see SI for experimental details), the sliding wall was translated with a micrometre translation stage until the membrane was located inside the purification channel (connecting wells 3 and 4) parallel to the preconcentration channel. The DNA plug was still located against the membrane after displacement (Fig. 5e). The preconcentrated DNA was then released in the Tris/TAPS/EDTA buffer by applying an electric field between reservoirs 3 and 4. The purified, released DNA plug can be observed in Fig. 5f in the downstream channel with a smaller section.

With these experiments, we demonstrated a new and simple way to integrate hydrogel membranes inside a microfluidic chip and to use them for preconcentration, purification and flow-free transport of macromolecules. To our knowledge, this is the first time that hydrogel membranes have been displaced inside a microfluidic system between several channels, and this possibility opens new routes for sample preparation and analytical operations. As the gel composition and polymerisation/gelation process of the integrated membrane can be modified at will, we envision that this technology can be used for trapping specific markers from samples using gel grafted with capture molecules. Elution could then be performed in a second channel for detection or dilution of the captured molecules in a clean and controlled buffer.

### Conclusions and perspectives

We report here the development of a new toolbox to expand the use of PDMS-based conventional microfluidics. The translation of rigid or semi-rigid sliding walls allowed valving, pumping, compartmentalisation, and various kinds of modifications of microfluidic networks. The sliding could also integrate additional features, such as microchannels or windows loaded with gels or solutions, increasing the potential of the technology beyond that of conventional in-chip valves. In this respect, the sliding wall can be seen as a one-dimensional version of Slip Chips, which also allows the relative motion of patterned microfluidic devices. Over the latter, sliding walls have the advantages of simpler operation and sustaining higher operation pressures at the cost of lower multiplexing power. Here, triggered “4D” cell migration and DNA purification and preconcentration were demonstrated as examples of functions achievable with a single sliding wall. Numerous others could be envisioned, in particular if several walls were actuated independently. Gas manipulation could also be envisioned using sliding switch valves via deflection of the flow. The use of on/off valves is not recommended for such an application, as pressurised gas would easily diffuse through PDMS when channels are blocked.

The integration of this new toolbox into an existing PDMS chip was straightforward and did not necessitate complex microfabrication. We envision that this simple, innovative and powerful technology will facilitate the spreading of microfluidics in non-microfluidics communities and be implemented for a large number of applications, especially low-cost, low-tech systems requiring only a few actuation events. Interestingly, we also demonstrated that sliding walls can be prepared by 3D printing, allowing for future versatile and low-cost implementation of walls with complex structures and functions libraries. The development of high-resolution 3D printing^34^ will accompany the development of sliding wall technology and expand the range of its applications.

For applications involving non-disposable chips, a systematic and quantitative study of the long-term evolution of the performance during a high number of cycles is required. Thus far, we did not observe changes in performance up to 50 actuations of the on/off valve. The limits of the technology in terms of operation speed will also require further investigation. For the moment, the translation time on the distances in our applications, typically 1 to 2 cm, involved approximately one second per operation, which is sufficient for applications involving single-use devices and sliding walls, for example, cell-based biology experiments and low-tech, low-cost systems.

Regarding automation, most applications demonstrated here were achieved with manual actuation or a simple translational stage with a resolution of typically 10 µm, showing the potential of the technology for low-cost, simple and manually operated devices. In recent years, however, the rapid development of low-cost robotics in various areas of industry and consumer products has stimulated the mass production of low-cost (typically from 10 to a few tens of €/US$), light weight (a few grams) and small size (typically for 1 to a few cm3) translation positioners, with position accuracy of 100 µm, which is quite sufficient for most applications of sliding walls. These devices operate at low voltage and can be controlled by compact and low cost processors and controllers (e.g., Arduino, Raspberry Pi), so the sliding wall technology is easily amenable to automation, while remaining much more compact and low-cost than other systems based, e.g., on pressure sources and/or “pinch” integrated valves.

## Materials and methods

### Chip microfabrication

All the microfluidic chips used in this study were made with PDMS. Sylgard 184 (Dow Corning, Midland, Michigan, USA) with a 10:1 base/curing agent ratio was poured and cured on brass moulds created by micro-milling (Minitech, Norcross, Georgia, USA). After curing, the PDMS replica was cut to open the guiding channels on each side of the PDMS chip, and reservoirs were punched. The replica was then bonded with a 1 min exposure to oxygen plasma (Cute, Femto Science, Gyeonggi-Do, South Korea) to a flat PDMS slab (thickness between 1 and 3 mm) to close the channels, and the PDMS slab was bonded to a glass slide. For the large compartmentalisation experiments, the flat PDMS slab was either replaced by a structured PDMS slab to provide guiding grooves in the PDMS roof and floor, either by a glass coverslip coated with a 140-µm-thick PDMS layer for the experiments including hydrogel integration. Incubation at 70 °C for 24 h after bonding restored the PDMS native hydrophobicity.

### Sliding wall microfabrication

Several materials and processes were used to fabricate sliding walls. Metallic sliding walls were cut into stainless steel sheets (100 µm thick) by micro-milling (Minitech, Norcross, Georgia, USA)). For the switch valve experiment, additional 50 µm-deep channels were engraved at the surface of the sliding wall with a 100 µm-diameter milling tool. The 3D-printed sliding walls were produced by stereolithography with a transparent resin (DS3000 from DWS systems, Thiene, Italy). Sliding walls for the pumping experiment were produced with a DigitalWax 028J+ printer (DWS Systems) with a 50-µm resolution. Sliding walls for the DNA preconcentration experiment were produced with a Dilase 3D (Kloé, Montpellier, France), a high-resolution stereolithographic printer providing a 5-µm resolution. NOA-based sliding walls were produced by filling a PDMS channel with NOA 81 glue (Norland Optical Adhesives, Cranbury, New Jersey, USA). This PDMS channel was formed by the superposition of two PDMS parts produced with a micro-milled mould containing a channel with a sliding wall shape and one perpendicular channel (see Supplementary Figure S1). A stainless-steel insert (5 mm × 250 µm × 100 µm), coated with a thin layer of liquid PDMS, was positioned in the perpendicular channels before superposition and alignment of the two PDMS parts. NOA 81 then filled the main channel by capillarity, followed by a 1 min exposure to UV (90 s at 30 mW/cm^2^, 365 nm). NOA structures were then removed from the PDMS mould, and the metallic insert was removed from the NOA sliding wall, creating a rectangular window. The sliding wall was exposed to a second UV illumination for 1 min. Hardening of the material was obtained by heating at 150 °C for 90 min.

### Pressure test experiments

The two types of valves produced were tested to determine the maximum pressure that can be applied before leakage and the optimal ratio between the guiding channel section and the sliding wall section. The chip inlet was connected with rigid tubing to a pressure controller (MFCS, Fluigent, Villejuif, France). Fluorescein solution was injected into the fluidic channel, and the liquid front was stopped close to the guiding channel. The sliding wall was then moved to close the valve. Fluorescein leakage along the walls was monitored with a fluorescence microscope, while pressure was increased by steps of 10 mbar until 1 bar. For the switch valve, the maximum flow rate tolerated was monitored using the same protocol, with the inlet linked to one outlet. The maximum flow rate was obtained by measuring the volume of liquid at the outlet after 1 min at the maximum pressure.

### Mature dendritic cell differentiation and migration in collagen gels

Mouse bone marrow-derived dendritic cells (BMDCs) were cultured for 10 days in BMDC medium (IMDM, FCS (10%), Glutamine (20 mM), Pen-Strep (100 U/ml) and 2-ME (50 µm)) supplemented with granulocyte-macrophage colony stimulating factor (50 ng/ml)-containing supernatant obtained from transfected J558 cells, as previously described^1^. Mature DCs were obtained by treating immature DCs with LPS (100 ng/ml) for 30 min and washing three times with BMDC medium. For collagen preparation, 120 µl of DCs (stock at 4 × 106 million/ml) were carefully mixed with 205 µl of bovine type I collagen (stock 3 mg/ml) (Advanced BioMatrix, San Diego, California) and 13 µl of NaHCO3 (stock 7.5%) (Merck, Darmstadt, Germany). All solutions were previously equilibrated at 4°C. The sample was then loaded in the PDMS chip and incubated at 37 °C for 30 min to allow collagen polymerisation. To generate the CCL21 gradient, BMDC medium containing 200 ng/ml of CCL21 was added to one side of the chamber. The cells were imaged by phase contrast at a frequency of 1 image every 2 min using a ×10 objective for 30 min as a control. The sliding wall was then removed, and the cells were imaged for 210 min. Images were processed to visualise cells by subtracting the mean image of the whole movie to every time point, obtaining white objects in a dark background. Then, the cells were tracked as previously described^36^.

### Preconcentration protocol

For the preconcentration and purification experiments, sliding walls with two windows were produced with high-resolution stereolithography. A drop of polyethylene-glycol diacrylate (PEGDA) hydrogel was deposited by pipetting in the window further to the wall tip (see Figure S4). The PEGDA solution was composed of 45% PEGDA 700, 3.5% Darocur (2-Hydroxy-2-methyl-1-phenyl-propan-1-one) and 51.25% 1X Tris-EDTA buffer. Photo-polymerisation was performed by exposure in an ozone cleaner for 2 min. The sliding walls were then stored in Tris-EDTA buffer before use.

The sliding wall was attached to a micrometre positioning stage using a homemade 3D-printed support to precisely control the motion of the sliding wall inside the guiding channel. The sliding wall was inserted inside the guiding channel, with the free window in the channel linking the reservoirs n°1 and n°2 (see Fig. 5a). TE 1X supplemented with SyBr Green I (10X) was loaded into the channel. The sliding wall was displaced to move the PEGDA membrane into this channel, and the empty window was placed in the second channel, linking the reservoirs n°3 and n°4. A second buffer consisting of 50 mM Tris, 50 mM Tris, and 2 mM EDTA was injected from the reservoir n°4. A total of 100 pg of Lambda-DNA (for the preconcentration experiment) or 100 ng of 25 bp DNA ladder from Invitrogen (for the release experiment) was loaded into the reservoir n°1. Platinum electrodes were plunged inside the reservoirs, and −20 and +20 V were applied to reservoirs n°1 and n°2, respectively. Pictures were taken with the FITC filter of an epifluorescence microscope. For the release experiment, the sliding wall was then moved after preconcentration to displace the membrane and the preconcentrated molecules to the second channel. In all, −500 and +500 V potentials were applied between reservoir n°4 and n°3, respectively.

Detailed chip dimensions, pictures of whole microchips and sliding walls and protocols for fluorescent collagen slab preparation and DNA preconcentration with an agarose membrane could be found in SI.

## Supplementary information

Supplementary Information

Graphical Abstract

Editoiral Summary

## Data Availability

The authors declare that the data supporting the findings of this study are available within the paper and its supplementary information files.
