# Supplementary material for: Sliding walls: a new paradigm for fluidic actuation and protocol implementation in microfluidics
Source: Microsyst Nanoeng. 2020 Apr 6;6:18. doi: 10.1038/s41378-019-0125-7 (PMC8433466; doi:10.1038/s41378-019-0125-7)
Supplement: Supplementary file 1 — Supplementary Information [file 41378_2019_125_MOESM1_ESM.docx]

**Supplementary information**

**Sliding walls: a new paradigm for fluidic actuation and protocol implementation in microfluidics**

Bastien Venzac^1,2,3^, Yang Liu^1,2,3^, Ivan Ferrante^1,2,3^, Pablo Vargas^3,4^, Ayako Yamada^1,2,3^, Rémi Courson^5^, Marine Verhulsel^1,2,3^, Laurent Malaquin^5^, Jean-Louis Viovy^1,2,3^*, Stéphanie Descroix^1,2,3^*

1 Laboratoire Physico Chimie Curie, Institut Curie, PSL Research University, CNRS UMR168, 75005 Paris, France

2 Sorbonne Universités, UPMC Univ Paris 06, 75005 Paris, France

3 Institut Pierre-Gilles de Gennes, PSL Research University, 75005 Paris, France

4 Institut Curie, PSL Research University, CNRS UMR 144, 75005 Paris, France

5 LAAS-CNRS, Université de Toulouse, CNRS, 31400 Toulouse, France

***** Equivalent contribution and corresponding authors

Corresponding authors: Jean-Louis Viovy: [jean-louis.viovy@curie.fr](mailto:jean-louis.viovy@curie.fr)

Stéphanie Descroix: [stephanie.descroix@curie.fr](mailto:stephanie.descroix@curie.fr)

**Detailed material and methods**

**Fabrication of NOA sliding wall**

**Fig. S1 a-b)** PDMS molds used for casting NOA sliding walls. After insertion of the metallic insert (see **a)**), the two molds are superimposed and NOA filled the channel by capillarity. **c)** NOA sliding wall after UV curing, removal from the PDMS mold and removing of the metallic insert. **d)** Side view of the window inside the NOA wall. Scale bars: 500 µm

**a)**

**b)**


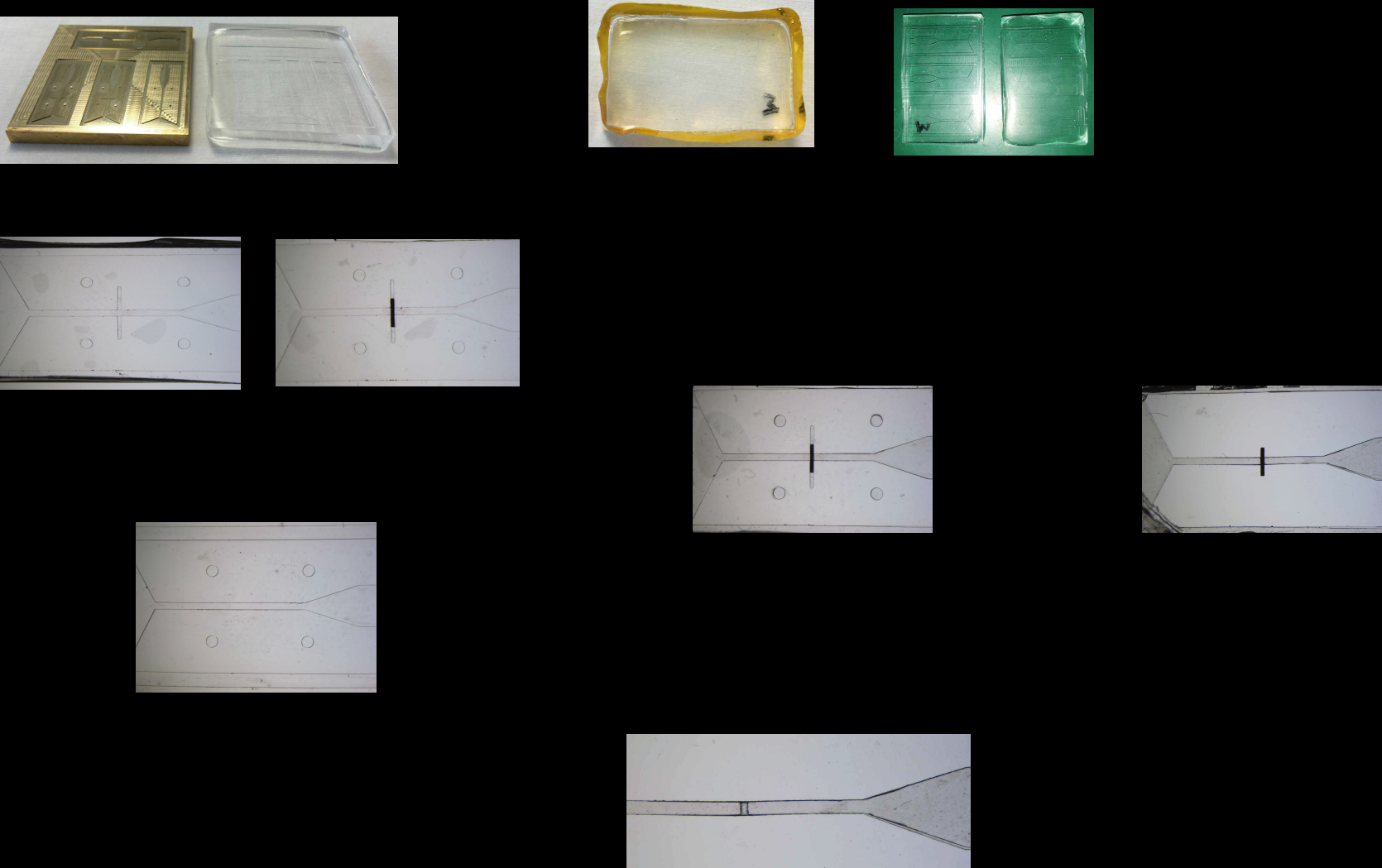

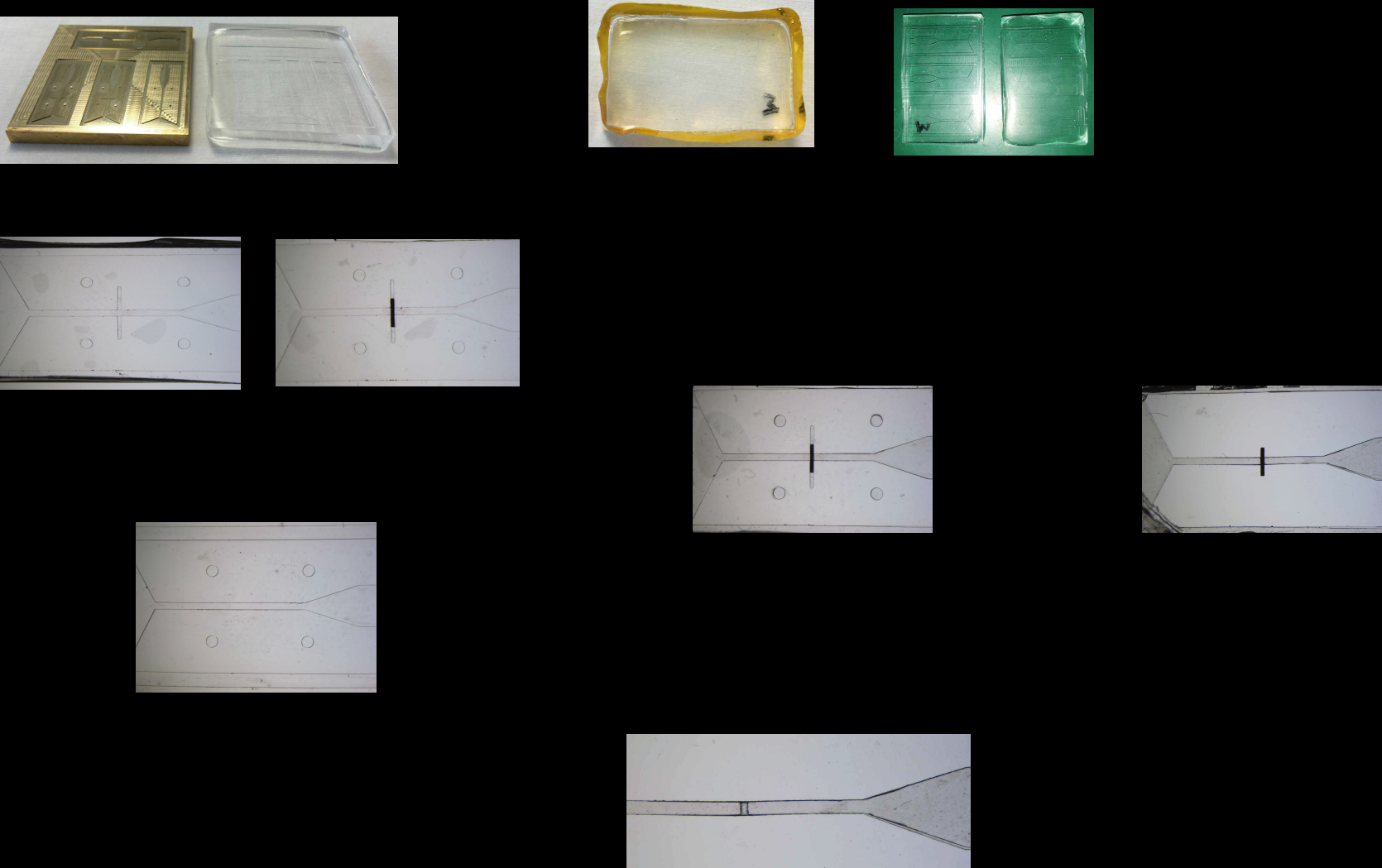

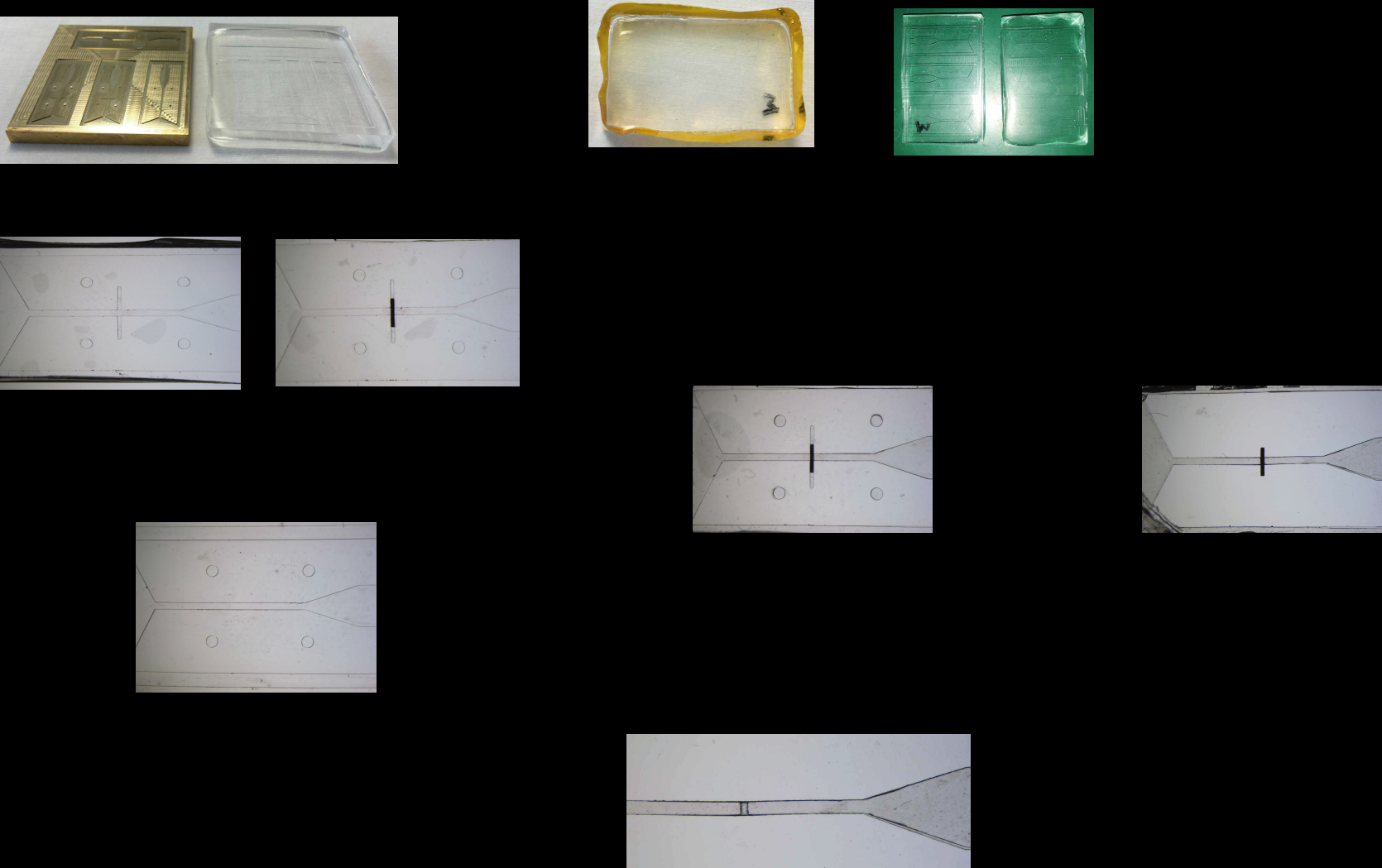

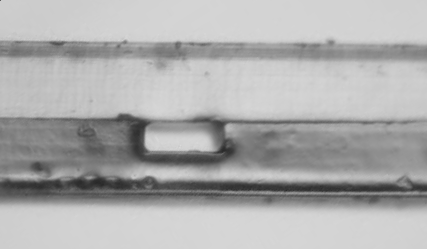


**c)**

**d)**

Metallic insert

**Complete design dimensions and whole chip pictures**


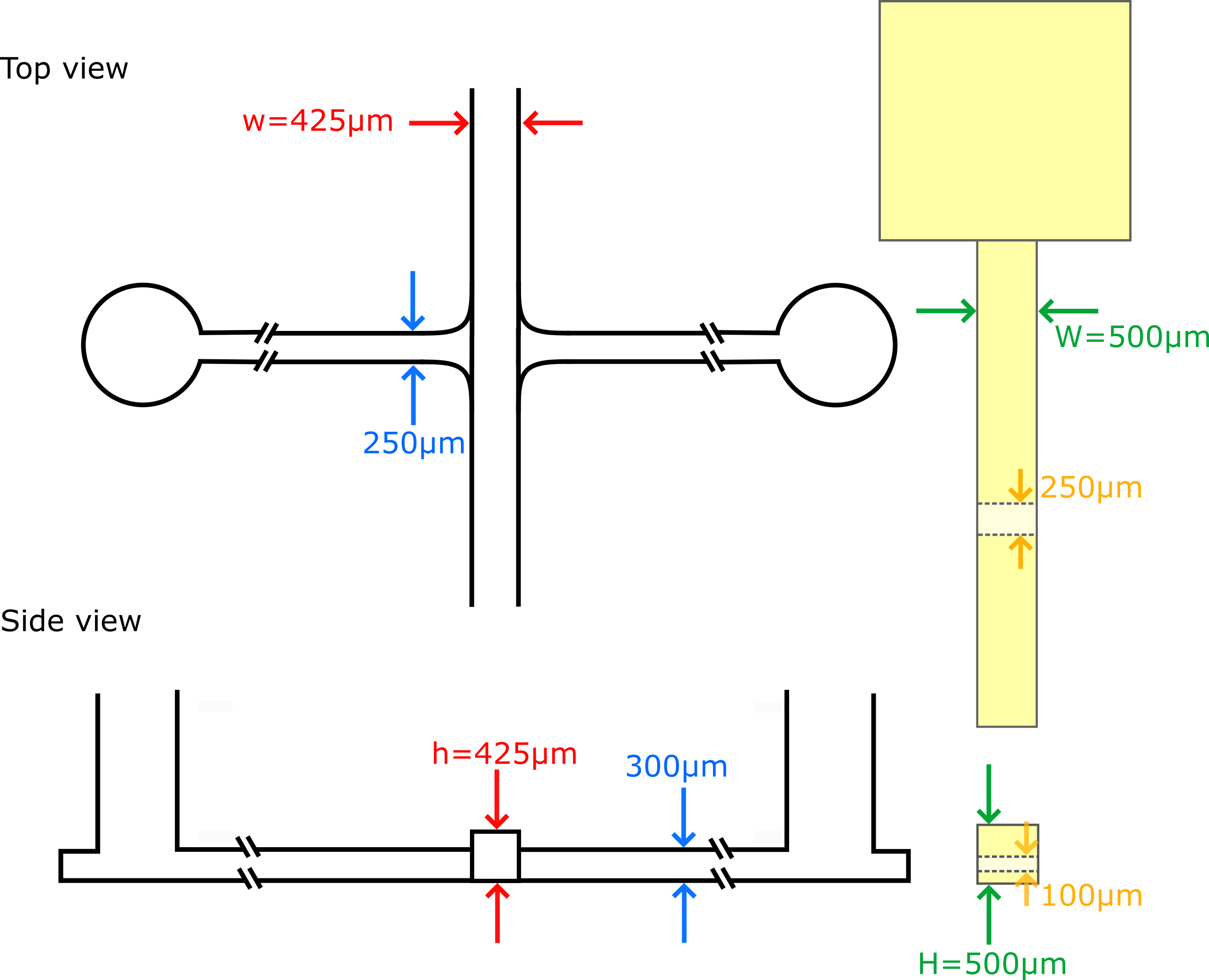

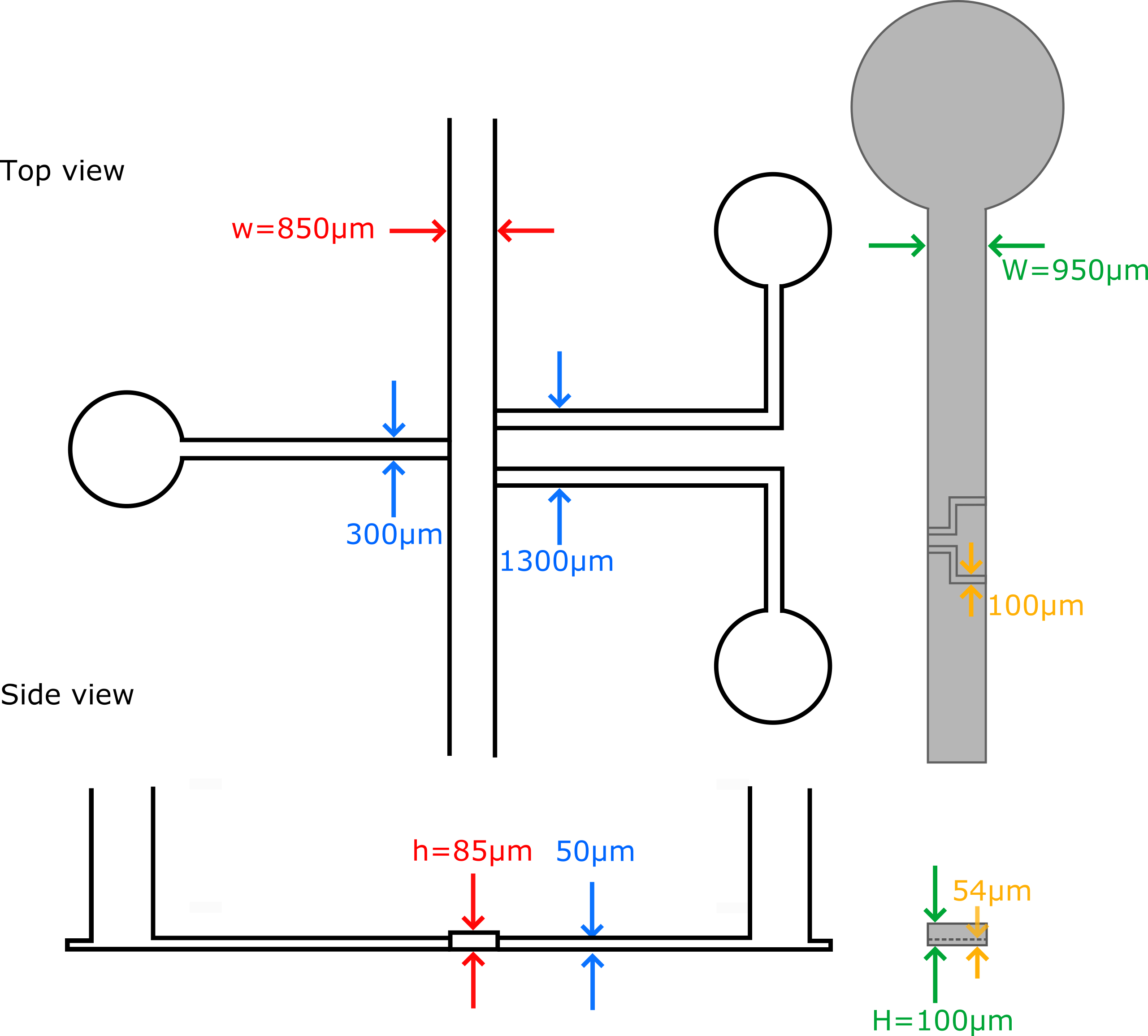


**Fig. S2** Dimensions of the designs for the valve experiments. a) Design of the chip (left) and NOA-based sliding wall (right) for the slide valve experiment. b) Design of the microchip (left) and the metallic sliding wall (right) for the switch valve experiment.

**a)**

**b)**

**Fig. S4** Design of the compartmentalization chip and sliding wall.


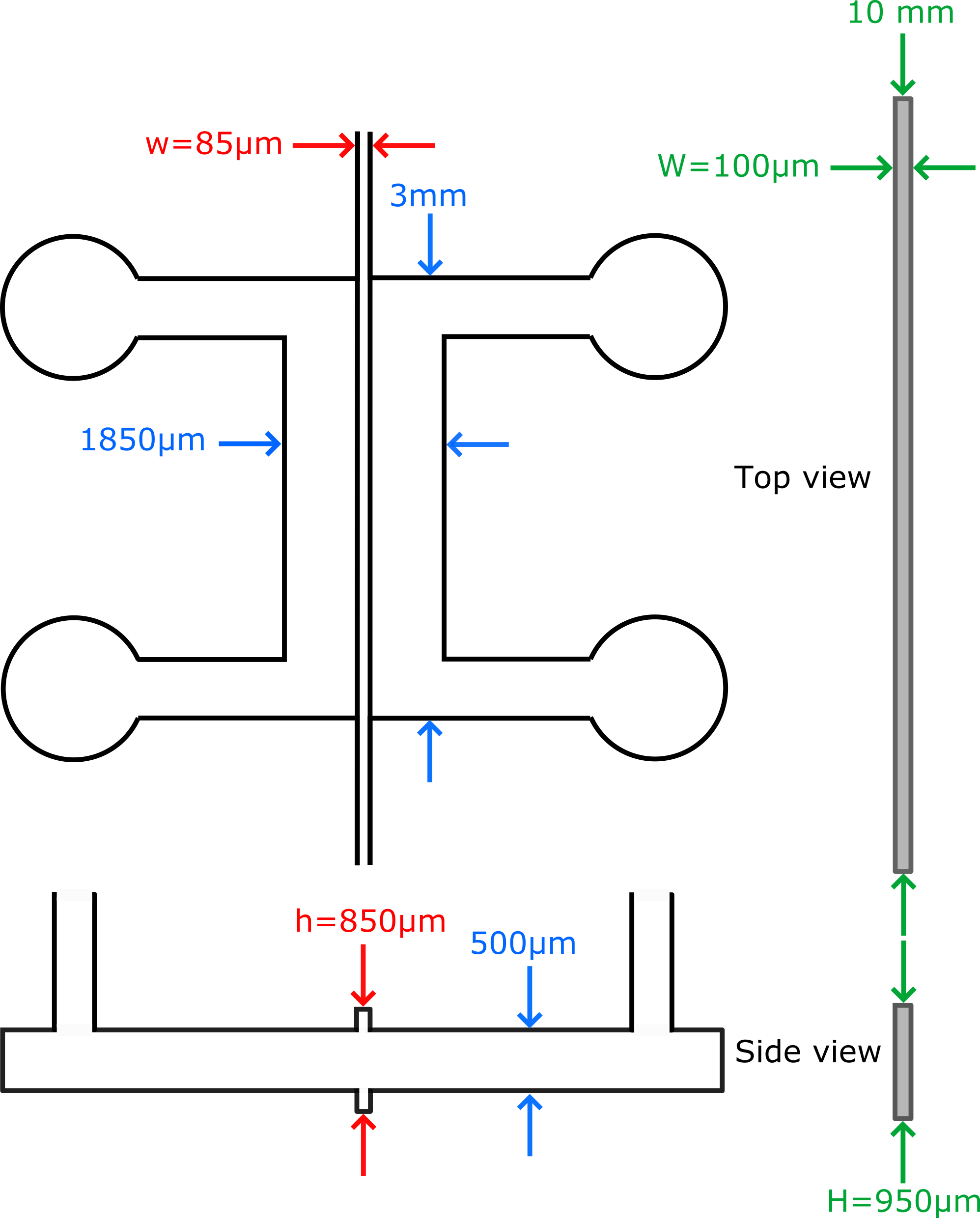

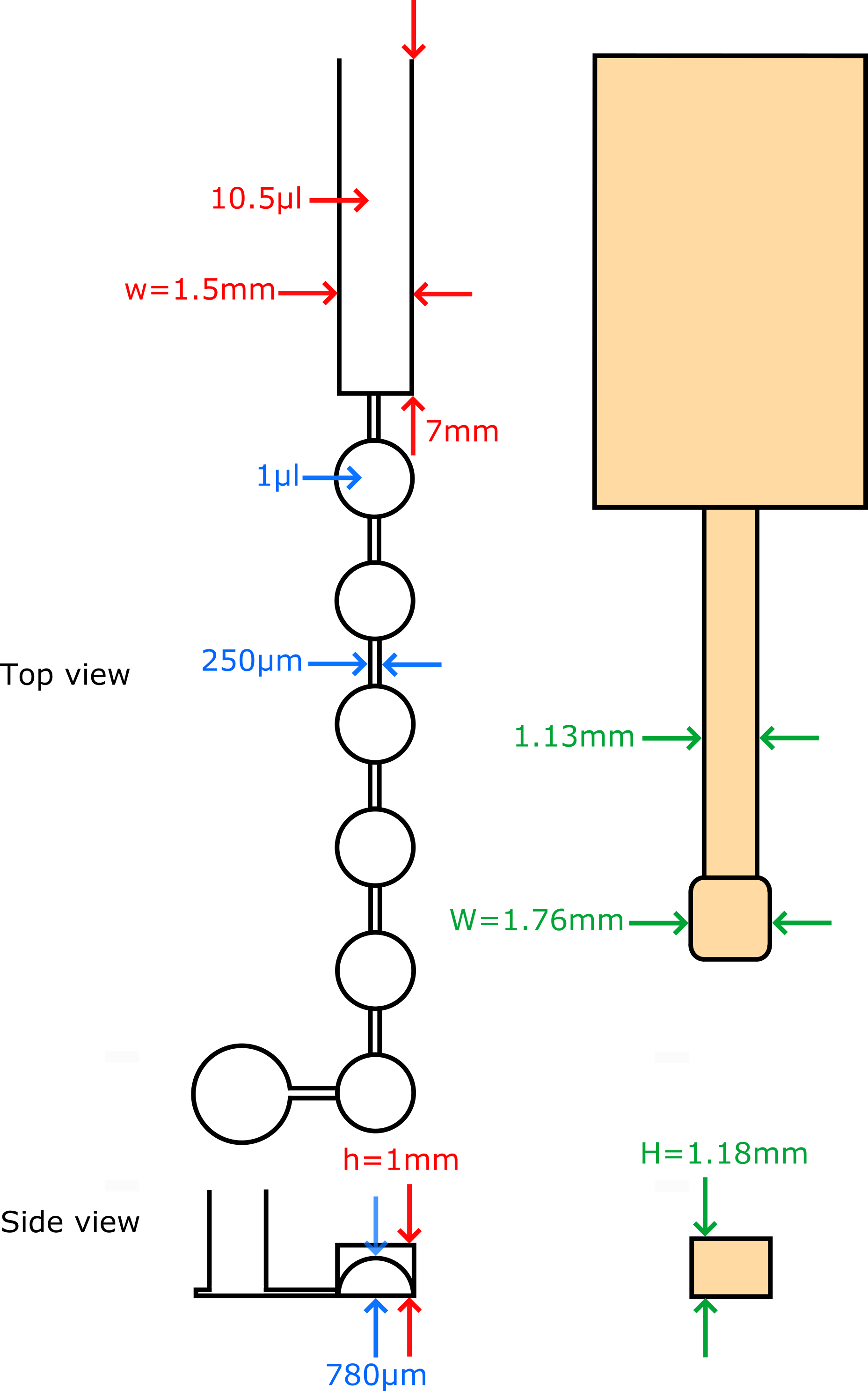


**Fig. S3** Design of the pumping chip and sliding wall.

**Fig. S7** Picture of the microchip and sliding wall for DNA preconcentration experiments. Blue and yellow dyes have been added for visualization.


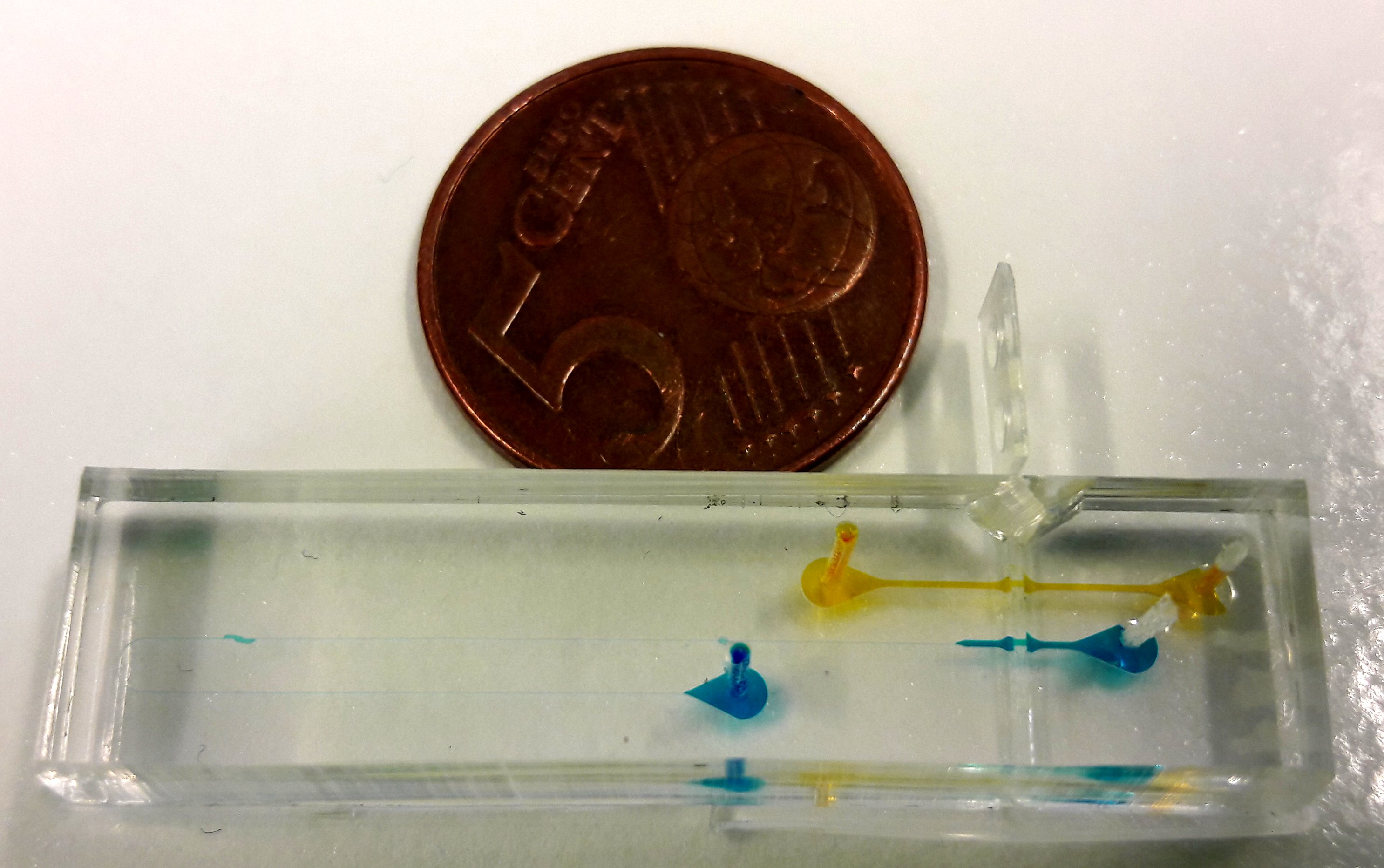


**Fig. S6** Picture of the microchip and sliding wall for compartmentalization experiments. Blue and yellow dyes have been added for visualization.


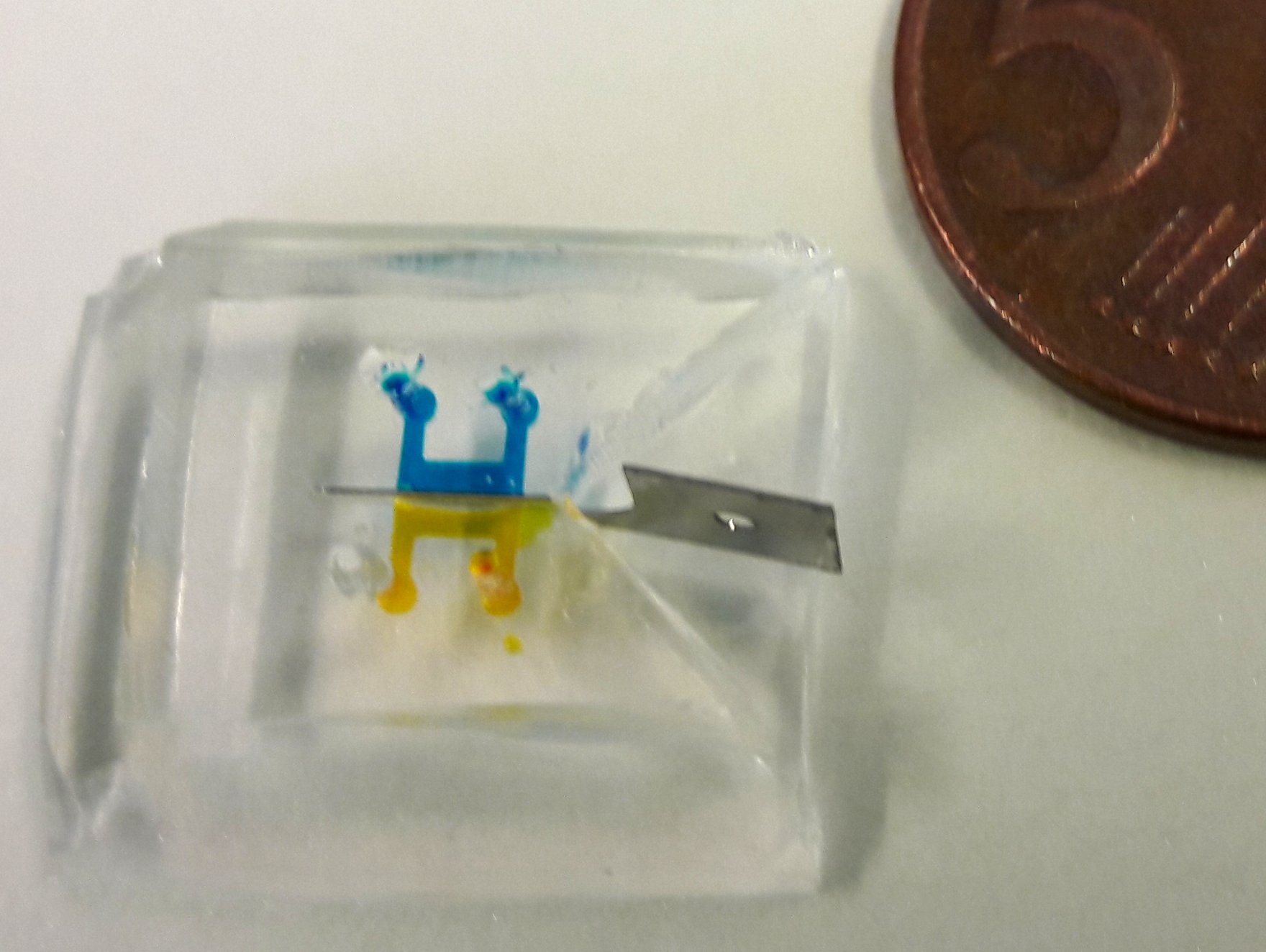


**Fig. S5** Design of the microchip and sliding wall for DNA preconcentration.


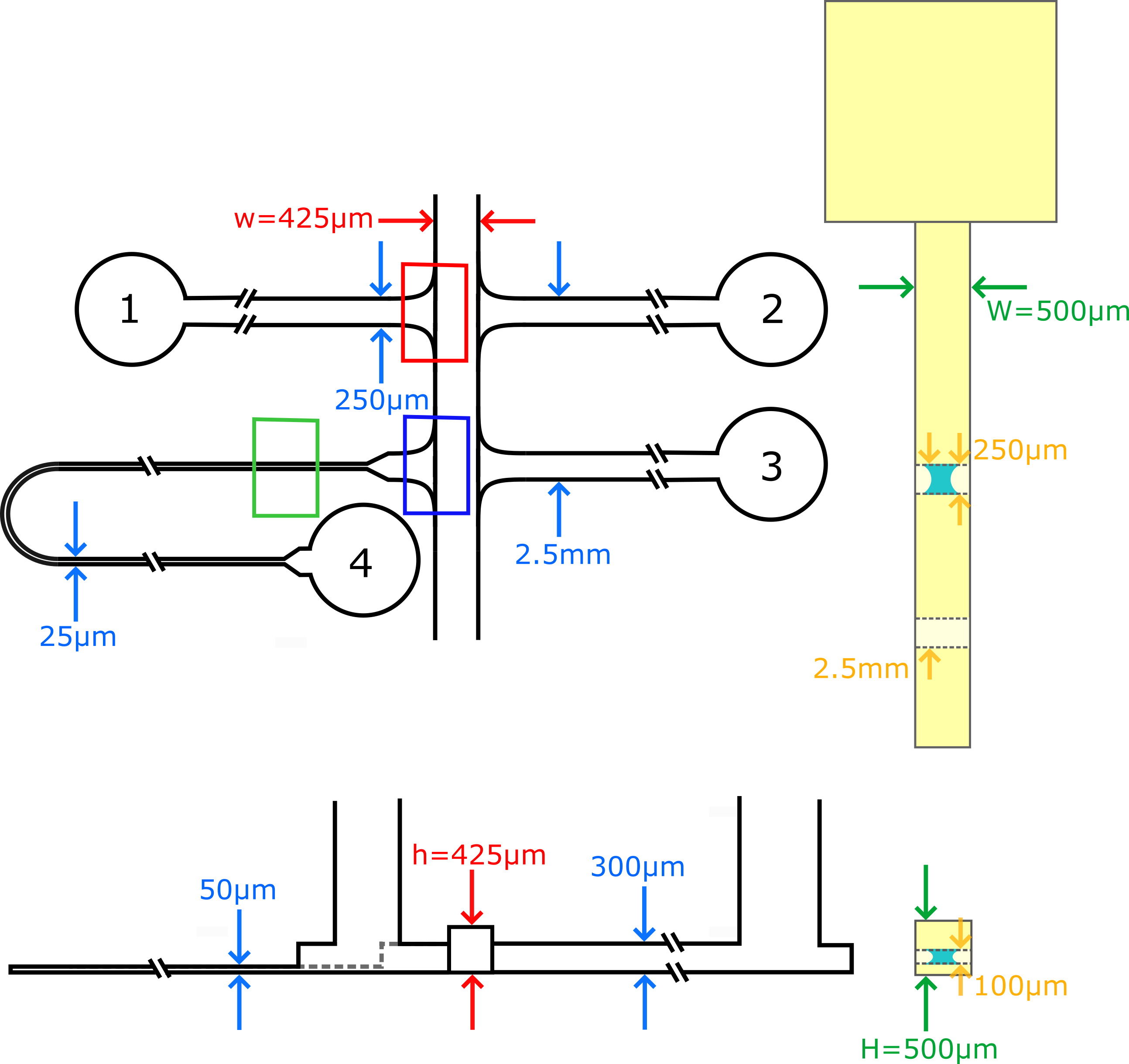


**Fig. S9** Picture of the microchip and sliding wall for on-chip pumping. Blue dye has been added for visualization.


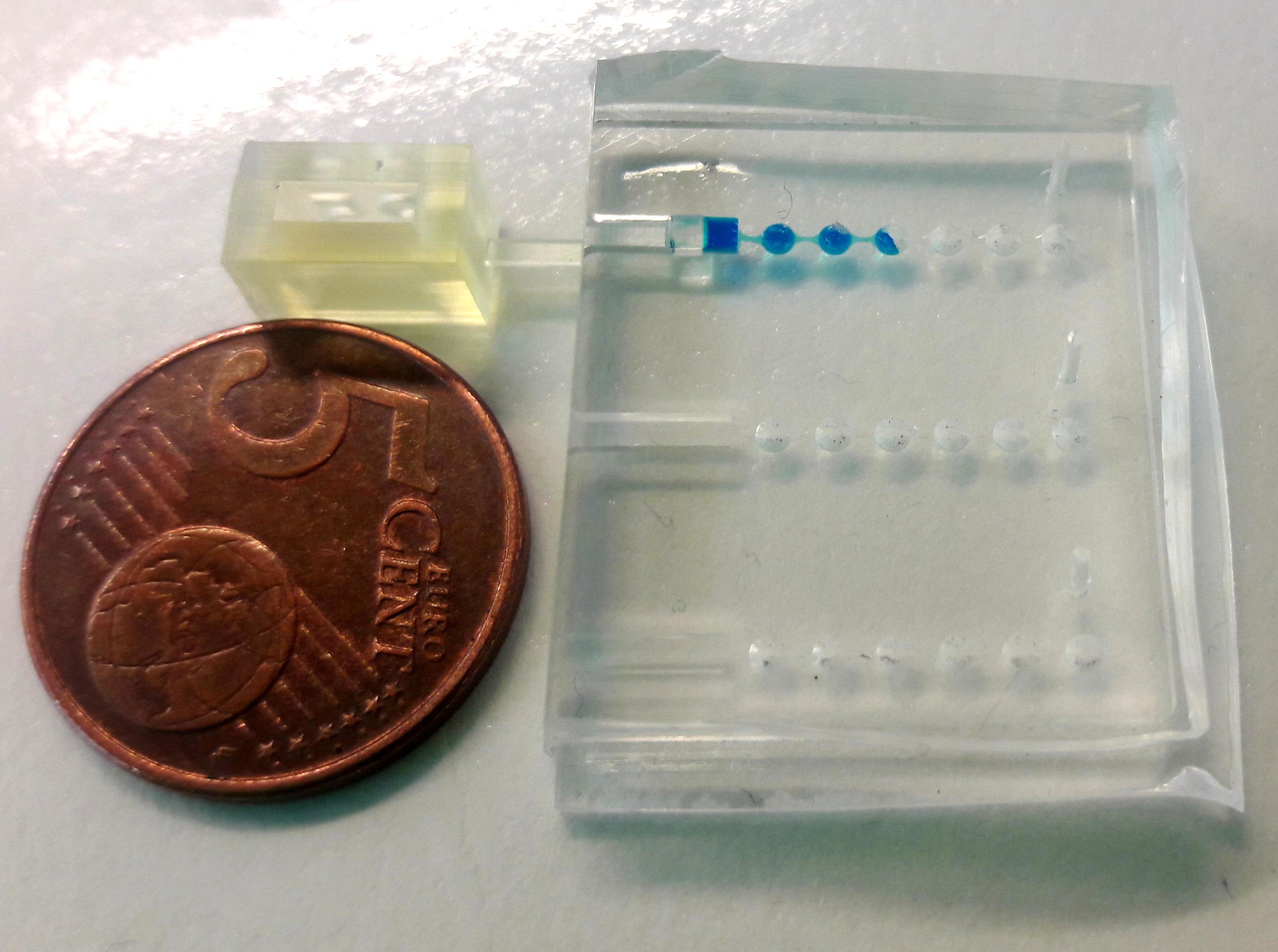


**Fig. S8** Picture of the microchip and sliding wall for on/off valving. Blue dye has been added for visualization.


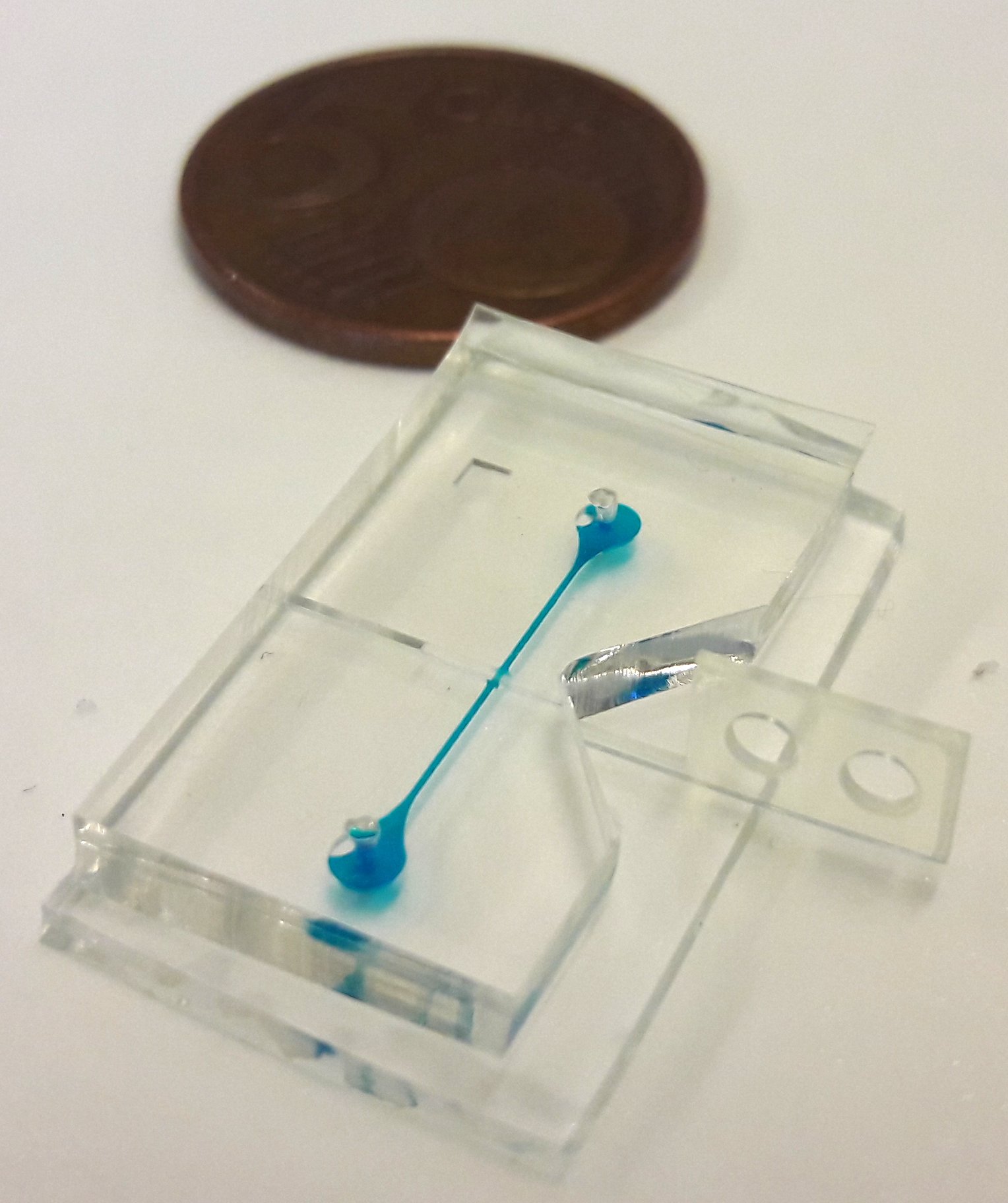


**Fluorescent collagen slab preparation and imaging**

Metallic sliding walls were incubated inside a 1% (w/w) bovine serum albumin solution (Sigma) during at least 40 min before insertion, to avoid collagen binding to the wall.

Rat tail collagen I (Corning, collagen I high concentration in 0.02N Acetic acid) was dialyzed overnight at 4°C against labelling buffer (0.25 M NaHCO3, 0.4 M NaCl, pH 9.5). Tetramethylrhodamine (TAMRA, Invitrogen) solution at 1 mg/mL in DMSO was prepared according to manufacturer’s instruction, added to collagen solution in (1:1) ratio and stirred overnight at 4°C. A subsequent overnight dialysis at 4°C against labelling buffer was performed to remove free dye from the labelled collagen solution. Labelled collagen was then dialysed overnight against in 0.02 N acetic acid solvent at 4°C. The TAMRA-labelled collagen stock solution was stored at 4°C and used at 1/20 dilution.

Rat tail collagen I (ThermoFisher) at 3mg/ml was mixed with the TAMRA-labelled collagen at a 20:1 ratio. pH was equilibrated to 7.0 with NaOH and PBS buffer. The mix was loaded into the first compartment of the microfluidic chip. PBS solution was loaded in the second half of the chamber. After 30 minutes at 37°C, the microfluidic chip was immerged into PBS to avoid collagen dehydration. After 2 h at 37°C, the sliding wall was slowly removed and the collagen slab was imaged with a TCS SP8 CSU confocal microscope (Leica).

**Preconcentration protocol with agarose membrane**

**Fig S10** Incorporation of an agarose membrane inside a sliding wall. a) Fluorescent observation of 3D-printed wall with a free windows inserted in a PDMS channel filled with 500 ng of SyBr-Green stained-Lambda-DNA, under electrophoretic motion from right to left. b) to d) Lambda-DNA accumulation in an agarose-filled window of a sliding wall over time.

500 µm


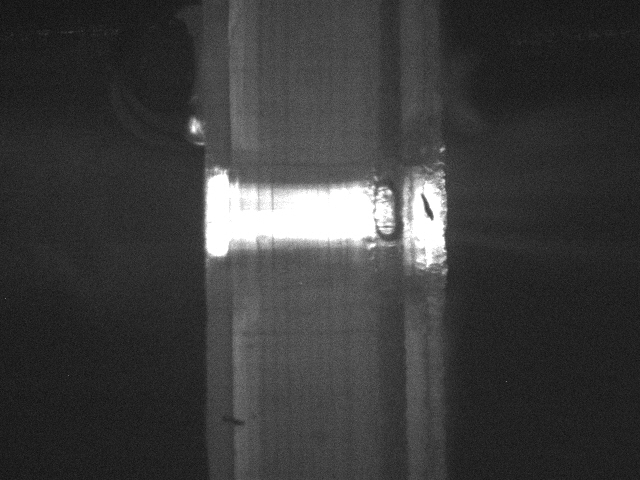

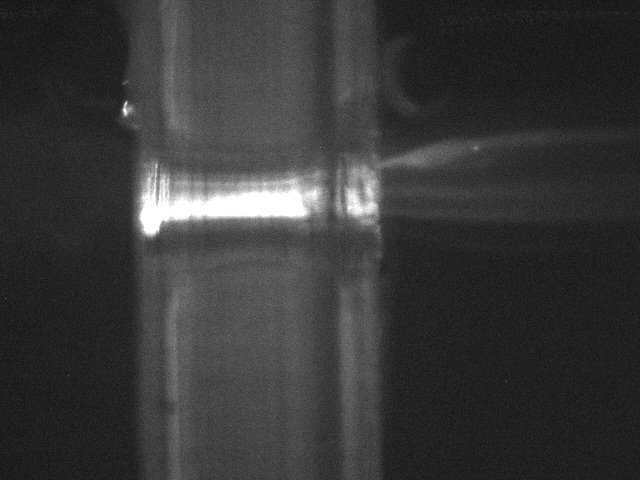

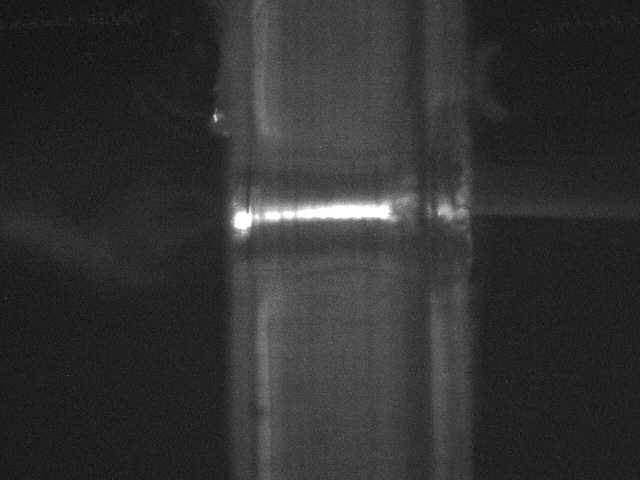

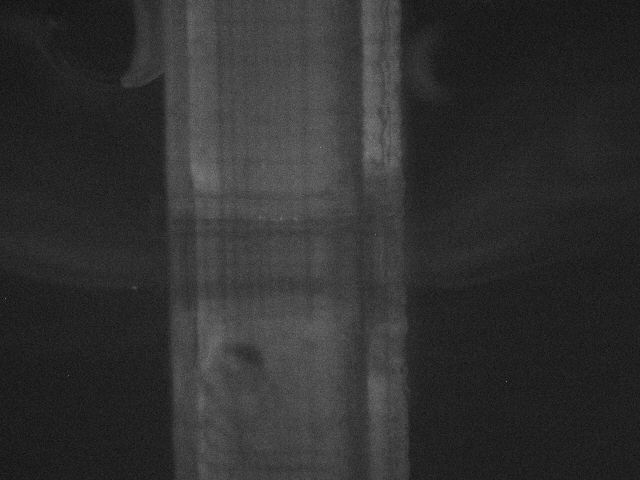


a)

b)

c)

d)

The sliding wall was inserted into the PDMS chip, with the window close to the base in the channel linking the reservoir 1 and 2. Then, the channel was filled with an agarose solution at 2.5% (w/w) in water just after boiling. To allow for membrane formation, the wall was moved to remove the agarose-filled window from the channel, and the whole chip was set at room temperature during 1 h for agarose gelling. The sliding wall was then removed from the chip, and inserted in another similar PDMS chip, for DNA preconcentration. Channel and reservoirs were filled with TE buffer containing 500 ng of Lambda-DNA stained with SyBr Green I. Agarose membrane was then moved into the channel, and a 100 V tension (supplied by a HVS448 system from LabSmith) was applied between two platinum electrodes immerged in the reservoirs 1 and 2. The DNA molecules migrated and were preconcentrated at the agarose membrane. As shown on Figure S5, the membrane was quickly saturated with fluorescent DNA molecules, entangled in the hydrogel mesh.

1. Vargas P, Maiuri P, Bretou M, Saez PJ, Pierobon P, Maurin M, Chabaud M, Lankar D, Obino D, Terriac E, et al.: Innate control of actin nucleation determines two distinct migration behaviours in dendritic cells. *Nat Cell Biol* 2016, 18:43-53.
