# Supplementary figures and images for: Sliding walls: a new paradigm for fluidic actuation and protocol implementation in microfluidics
Source: Microsyst Nanoeng. 2020 Apr 6;6:18. doi: 10.1038/s41378-019-0125-7 (PMC8433466; doi:10.1038/s41378-019-0125-7)

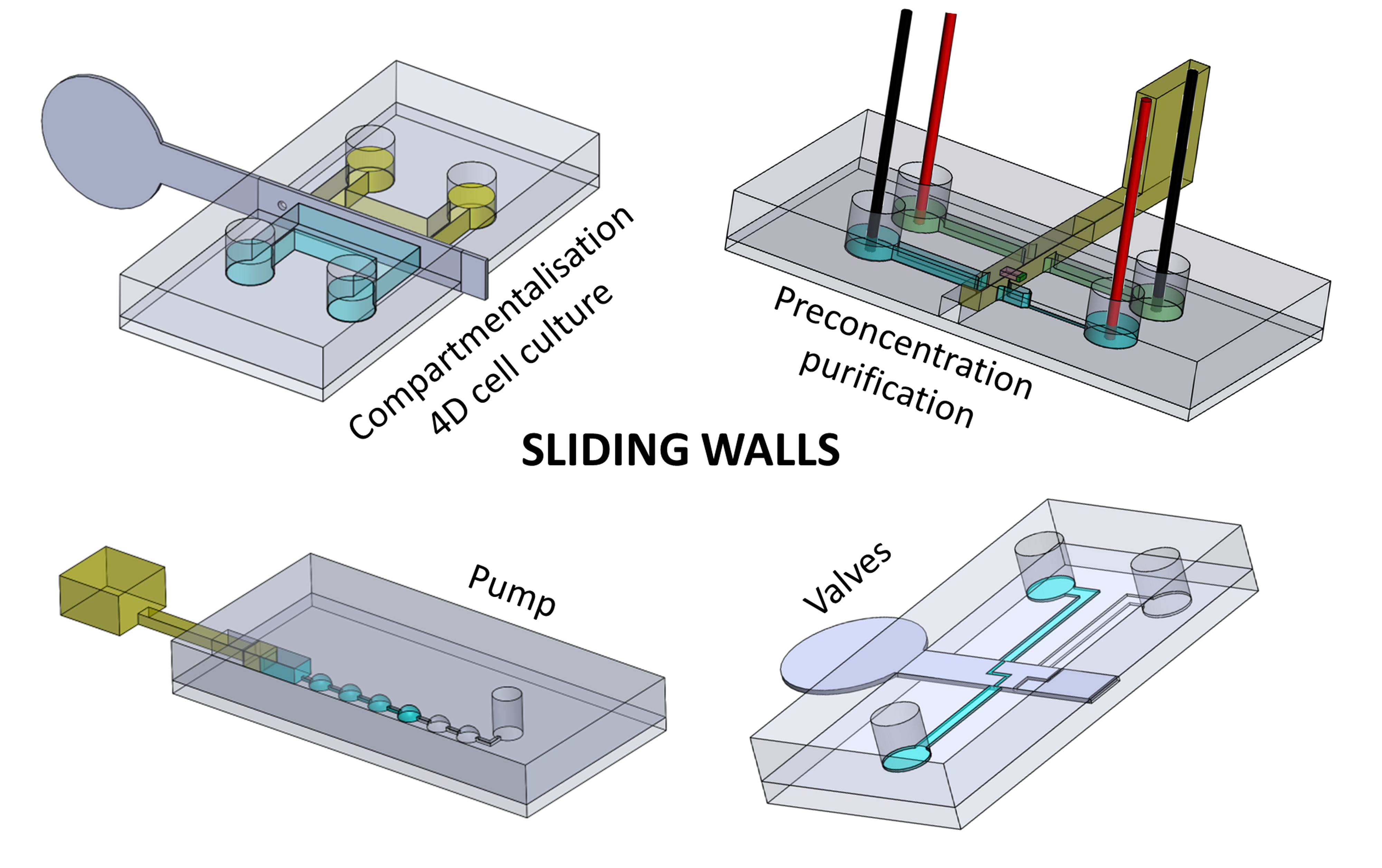

Supplement: Supplementary file 2 — Graphical Abstract [file 41378_2019_125_MOESM2_ESM.tif]
